# Optical Force on a Metal Nanorod Exerted by a Photonic Jet

**DOI:** 10.3390/nano12020251

**Published:** 2022-01-13

**Authors:** Bojian Wei, Shuhong Gong, Renxian Li, Igor V. Minin, Oleg V. Minin, Leke Lin

**Affiliations:** 1 School of Physics and Optoelectronic Engineering, Xidian University, Xi’an 710071, China; bjwei@stu.xidian.edu.cn (B.W.); shgong@xidian.edu.cn (S.G.); 2 Collaborative Innovation Center of Information Sensing and Understanding, Xidian University, Xi’an 710071, China; 3 School of Nondestructive testing, Tomsk Polytechnic University, 634050 Tomsk, Russia; ivminin@tpu.ru (I.V.M.); ovminin@tpu.ru (O.V.M.); 4 China Research Institute of Radiowave Propagation, Qingdao 266000, China; llk22s@163.com

**Keywords:** optical force, photonic jet, nanorod, dipole approximation, Generalized Luneburg Lens

## Abstract

In this article, we study the optical force exerted on nanorods. In recent years, the capture of micro-nanoparticles has been a frontier topic in optics. A Photonic Jet (PJ) is an emerging subwavelength beam with excellent application prospects. This paper studies the optical force exerted by photonic jets generated by a plane wave illuminating a Generalized Luneburg Lens (GLLs) on nanorods. In the framework of the dipole approximation, the optical force on the nanorods is studied. The electric field of the photonic jet is calculated by the open-source software package DDSCAT developed based on the Discrete Dipole Approximation (DDA). In this paper, the effects of the nanorods’ orientation and dielectric constant on the transverse force *F_x_* and longitudinal force *F_y_* are analyzed. Numerical results show that the maximum value of the positive force and the negative force are equal and appear alternately at the position of the photonic jet. Therefore, to capture anisotropic nanoscale-geometries (nanorods), it is necessary to adjust the position of GLLs continuously. It is worth emphasizing that manipulations with nanorods will make it possible to create new materials at the nanoscale.

## 1. Introduction

Optical capture by manipulating neutral atoms by lasers was first reported by Ashkin et al. in 1970 [1]. At present, optical manipulation can achieve the capture of living cells and organelles [2], DNA rotation [3], and chromosome surgery [4] with low damage, which is very important in modern medicine and biology. Later, optical capture was gradually applied to the manipulation of various nanomaterials. Nanomaterials and nanotechnology are already indispensable and important members of modern technology. For example, quantum dots play an important role in fluorescence detection, bioluminescence detection and other fields due to their unique photoelectric properties [5]. Semiconductor nanowires [6] have excellent performance in electronics and photonics due to their tunable direct bandgap and high carrier mobility. Anisotropic nanoscale-geometries, including nanorods [7], can be used in molecular imaging and photothermal cancer therapy. The capture and manipulation of nanomaterials are significant in the optoelectronic industry. Different arrangements of nanomaterials have different optical and physical properties [8], and optical force is an important way to realize the complex arrangement structure of nanomaterials. In 2006, Pelton et al. [9] reported three-dimensional optical capture of gold nanorods and enhanced optical force by longitudinal surface plasmon resonance. In 2008, Selhuber-Unkel [10] performed a quantitative analysis of the optical force on gold nanorods. The results showed that the interaction between an optical trap and a nanorod is related to the particle’s polarizability. In 2010, Tong et al. [11] used a linearly polarized near-infrared laser to arrange and rotate nanostructures, including nanorods, optically. In 2011, Ruijgrok et al. [12] quantitatively measured the torque exerted on the nanorod. In 2014, Liaw et al. [13] studied the polarizability of gold nanorods and analyzed the optical torque under the Maxwell stress tensor. In 2017, Fick et al. [14] captured nanorods using optical fiber nanotweezers. In 2020, Huang et al. [15] studied the capture of gold nanorods by a plasmonic tweezer. By adjusting the mode of local surface plasmon resonance, the force direction can be changed. Capturing nanorods and arranging them according to different patterns can enable nanoarrays to have new functions. In addition, the dipole approximation is a common and accurate method to study optical force. In this article, we use the dipole approximation method to numerically simulate the optical force exerted by the photonic jet on the nanorods.

When a beam of light illuminates a particle (its size is close to the wavelength), the backlit side of the particle will generate a strong and narrow beam. Scholars vividly named it a photonic jet. Photonic jet has been used in many fields. For example, optical data storage [16], superresolution imaging [17], and Raman signal enhancement [18], etc. At the same time, a photonic jet, as a highly focused beam, can also be used as a light source to capture and manipulate particles [19]. Its application in cell surgery [20,21,22] proves this point. In this paper, we will generate PJ by illuminating a mesoscale Generalized Luneburg Lens with a plane wave, which, however, does not reduce the generality of the problem, and the results can be generalized to other particle-lens combinations. GLLs is the particle model mentioned in Mao et al.’s [23] paper. The refractive index of this model is rotationally symmetrical and can generate an ultra-narrow or ultra-long PJ. This PJ is of great significance in the optical trapping of nanoparticles. We can change PJ significantly by changing the wavelength, focal length, and radius of GLLs, and then analyze the influence of PJ on the optical force.

PJ and optical force are studied in the framework of Discrete Dipole Approximation [24]. The basic idea of DDA comes from DeVoe’s writings. DDA has studied scattering by first dividing particles of any shape into dipole arrays (polarizable) whose size is negligible compared to the wavelength [25]. Then the interaction between the dipoles and the incident field is analyzed to solve the whole field after the scattering. Until today, DDA has been developed into a numerical simulation method of the electromagnetic field with high precision and fast operation speed. In particular, DDSCAT [26] is an open-source software package of DDA with simple operation methods and accurate calculation results. DDSCAT is especially useful for a particle with a special shape. The coordinate system for the dipole array inside the particle in DDSCAT is straightforward to understand and modify. When calculating the scattering of a particle with an irregular shape, we only need to find the surface function and combine it with the dipole coordinate system [27]. Therefore, DDSCAT is software suitable for calculating the scattered field of GLLs. Dipole approximation [28] is a common method for numerical simulating the optical force exerted on nanostructures. In this calculation model, the nanostructure is regarded as a dipole (the size is almost negligible). In the calculation of optical force, the shape of the nanostructure can be expressed by its polarizability. The polarizability of a nanoparticle with a more complex shape is often a tensor [29]. The expression of the polarizability of the nanorod in this article will be shown in the second section.

The rest of this paper is distributed as follows. The second section introduces the theory of DDA and DDSCAT to calculate the near-field scattering, and then analyzes the theory of force on nanorod in the dipole approximation framework. In the third section, the numerical results of transverse and longitudinal forces on nanorods and the effects of wavelength, focal length, and radius of GLLs on the optical forces are discussed. The fourth section summarizes the full article.

## 2. Materials and Methods

### 2.1. Discrete Dipole Approximation

In the above, we briefly introduced the method of DDA to calculate near-field scattering. In this section, we will analyze the principle of DDA in detail. The electric dipole moment of a polarizable (the polarizability is $\alpha_{i}$) dipole is $P_{i}=\alpha_{i}E_{i}$ [30]. The total electric field at $r_{i}$ in the calculation area can be expressed as [31]:

(1) $$E_{i}=E_{inc,i}+E_{sca,i}$$

where $E_{inc,i}$ and $E_{sca,i}$ are the incident electric field and scattered electric field at $r_{i}$. $-A_{ij}P_{j}$ represents the scattering field generated by the dipole (at $r_{j}$) at $r_{i}$. Therefore, Equation (1) can be expressed as:

(2) $$E_{i}=E_{inc,i}-\sum_{j\neq i}A_{ij}P_{j}$$

where, $A_{ij}$ [32] represents the influence of the dipole at position $r_{j}$ on the dipole at position $r_{i}$. $A_{ij}=\frac{\exp\left(ikr_{ij}\right)}{r_{ij}}\times \left[k^{2}\left({\hat{r}}_{ij}{\hat{r}}_{ij}-1_{3}\right)+\frac{ikr_{ij}-1}{r_{ij}^{2}}\left(3{\hat{r}}_{ij}{\hat{r}}_{ij}-1_{3}\right)\right],i\neq j$, where, k is the wave vector, $r_{ij}$ is the distance between point i and point j, and ${\hat{r}}_{ij}$ is the unit vector from i to j. $1_{3}$ is the identity matrix. ${\hat{r}}_{ij}{\hat{r}}_{ij}$ and $1_{3}$ are shown in Equations (3) and (4) [33,34]. $r_{x}$, $r_{y}$, and $r_{z}$ are the coordinate components of ${\hat{r}}_{ij}$ along the x, y, and z directions, respectively. $\sum_{j\neq i}A_{ij}P_{j}$ represents the scattered field at that point:

(3) $${\hat{r}}_{ij}{\hat{r}}_{ij}=\left[\begin{array}{ccc} r_{x}^{2} & r_{x}r_{y} & r_{x}r_{z}\\ r_{y}r_{x} & r_{y}^{2} & r_{y}r_{z}\\ r_{z}r_{x} & r_{z}r_{y} & r_{z}^{2} \end{array}\right]$$

(4) $$1_{3}=\left[\begin{array}{lll} 1 & 0 & 0\\ 0 & 1 & 0\\ 0 & 0 & 1 \end{array}\right]$$

Now, we can get the electric dipole moment at any position [35,36]:

(5) $$P_{i}=\alpha_{i}(E_{inc,i}-\sum_{j\neq i}A_{ij}P_{j})$$

(6) $$E_{inc,i}=\sum_{j\neq i}A_{ij}P_{j}+\alpha_{i}^{-1}P_{i}$$

where [37]:

(7) $$\begin{array}{l} \alpha_{i}\approx \frac{\alpha^{CM}}{1+\left(\alpha^{CM}/d^{3}\right)\left[\left(b_{1}+m^{2}b_{2}+m^{2}b_{3}S\right){\left(kd\right)}^{2}-(2/3)i{\left(kd\right)}^{3}\right]},\\ b_{1}=-1.891531,b_{2}=0.1648469,\\ b_{3}=-1.7700004,S\equiv \sum_{i=1}^{3}{\left({\hat{a}}_{i}{\hat{e}}_{i}\right)}^{2}, \end{array}$$

(8) $$\alpha_{i}^{CM}=\frac{3d^{3}}{4\pi }\frac{n_{i}-1}{n_{i}+2}$$

where, $\alpha_{i}^{CM}$ is the Clausius-Mossotti polarizabilities (as shown in Equation (8)), m and $n_{i}$ are the refractive index and the dielectric constant at any position. d is the distance between dipoles, $b_{1}$, $b_{2}$, and $b_{3}$ are constants, k is the wave vector, $\hat{a}$ and $\hat{e}$ are the unit vectors of the incident direction and the polarization direction [38].

In this paper, $n_{i}=n_{0}{\left[1+f_{GLLs}^{2}-{\left(r_{0}/R\right)}^{2}\right]}^{1/2}/f_{GLLs}$, where, $n_{0}=1$. $f_{GLLs}$ are the focal length normalized radii of GLLs. It is unitless. $r_{o}$ is the radial coordinate (o = 1, 2, 3......, 30) and R is the maximum radius of the GLLs. Due to the limitation of memory and computing speed, we only divide the GLLs into 30 layers, and we will verify the correctness of this model in Section 3.1. The design method and correctness verification of GLLs in DDSCAT have been completed in our previous work [39], and will not be described in this paper. Defining $A_{jj}\equiv \alpha_{j}^{-1}$, Equation (6) has the following form [40]:

(9) $$E_{\mathrm{inc},\;i}=\sum_{j\in \;\mathrm{target}\;}A_{ij}P_{j}$$

In DDSCAT, the inner (original target sites j) and outer (vacuum sites i) fields of a particle will be represented separately as [41]:

(10) $$E=E_{inc}+E_{scat}=\{\begin{array}{cc} \alpha_{j}^{-1}P_{j} & \;\mathrm{original}\;\mathrm{target}\;\mathrm{sites}\;j\\ E_{inc,i}-\sum_{j\in t\arg et}A_{ij}P_{j} & \;\mathrm{vacuum}\;\mathrm{sites}\;i \end{array}$$

Finally, the electric dipole moment outside the particle is 0.

### 2.2. Optical Force on a Nanorod

In the framework of the dipole approximation, the polarizability of arbitrarily oriented nanorod is put into the optical force equation as follows [42]:

(11) $$\alpha_{z}=V\varepsilon_{d}\frac{\varepsilon_{m}-\varepsilon_{d}}{\varepsilon_{d}}$$

(12) $$\alpha_{t}=2V\varepsilon_{d}\frac{\varepsilon_{m}-\varepsilon_{d}}{\varepsilon_{m}+\varepsilon_{d}}$$

where, V is the volume of nanorods, $\varepsilon_{m}$ and $\varepsilon_{d}$ are the dielectric functions of metal and medium, respectively. The polarizability tensor of the particle is [29]:

(13) $$\hat{\alpha }=\left|\begin{array}{ccc} \alpha_{z} & 0 & 0\\ 0 & \alpha_{t} & 0\\ 0 & 0 & \alpha_{t} \end{array}\right|$$

**Figure 1:**
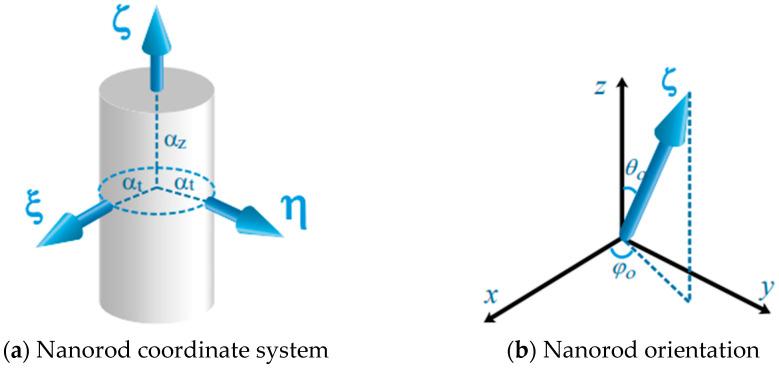
(**a**,**b**) respectively show the nanorod’s coordinate system (ξ,η,ζ) and the angle between the axis (ξ) of the nanorod and the (x,y,z) coordinate system ($\theta_{0},\varphi_{0}$). $\theta_{0}$ represents the angle between the axis of the nanorod and the z-axis, and $\varphi_{0}$ represents the angle between the projection of the axis of the nanorod on the xoy plane and the x-axis.

The coordinate system inside the particle is (ξ,η,ζ). The schematic diagram of the polarization distribution and orientation of the nanorods is shown in Figure 1. $\alpha_{z}$ and $\alpha_{t}$ represent the longitudinal (parallel to the axis) and transverse (parallel to the bottom surface) polarizability of the nanorod, respectively. Both the ξ and η axes are parallel to the bottom surface of the nanorod, so the polarizability along these two directions is the same. The rotation matrix connecting the two systems is [29,43]:

(14) $$\hat{R}\left(\theta_{0},\varphi_{0}\right)=\left(\begin{array}{ccc} \cos\varphi_{0}\cos\theta_{0} & -\sin\varphi_{0} & \cos\varphi_{0}\sin\theta_{0}\\ \sin\varphi_{0}\cos\theta_{0} & \cos\varphi_{0} & \sin\varphi_{0}\sin\theta_{0}\\ -\sin\theta_{0} & 0 & \cos\theta_{0} \end{array}\right)$$

The polarizability tensor of the rotated nanorod in the system of coordinates xyz is then done by the matrix product [44]:

(15) $$\hat{A}={\hat{R}}^{-1}\hat{\alpha }\hat{R}$$

and the nanorod dipole moment p excited by the total electric field E at the nanorod position r=(x,y,z) is:

(16) $$p=\hat{A}\left(\theta_{0},\varphi_{0}\right)E(x,y,z)$$

Finally, the optical force on the nanorods is [45]:

(17) $$F_{\xi }=\frac{1}{2}ℜ\left\{p\cdot \partial_{\xi }E^{*}\right\},\xi =x,y,z$$

By rewriting p as:

(18) $$p=\left[\begin{array}{l} p_{1}\\ p_{2}\\ p_{3} \end{array}\right]$$

Equation (17) can be expanded to:

(19) $$\begin{array}{l} F_{x}=\frac{1}{2}ℜ\left\{p_{1}\cdot \frac{\partial E_{x}^{*}}{\partial x}+p_{2}\cdot \frac{\partial E_{y}^{*}}{\partial x}+p_{3}\cdot \frac{\partial E_{z}^{*}}{\partial x}\right\}\\ F_{y}=\frac{1}{2}ℜ\left\{p_{1}\cdot \frac{\partial E_{x}^{*}}{\partial y}+p_{2}\cdot \frac{\partial E_{y}^{*}}{\partial y}+p_{3}\cdot \frac{\partial E_{z}^{*}}{\partial y}\right\}\\ F_{z}=\frac{1}{2}ℜ\left\{p_{1}\cdot \frac{\partial E_{x}^{*}}{\partial z}+p_{2}\cdot \frac{\partial E_{y}^{*}}{\partial z}+p_{3}\cdot \frac{\partial E_{z}^{*}}{\partial z}\right\} \end{array}$$

In this paper, we will analyze the $F_{x}$, longitudinal force $F_{y}$ and $F_{z}$ exerted by the photonic jet on a nanorod.

## 3. Results

**Figure 2:**
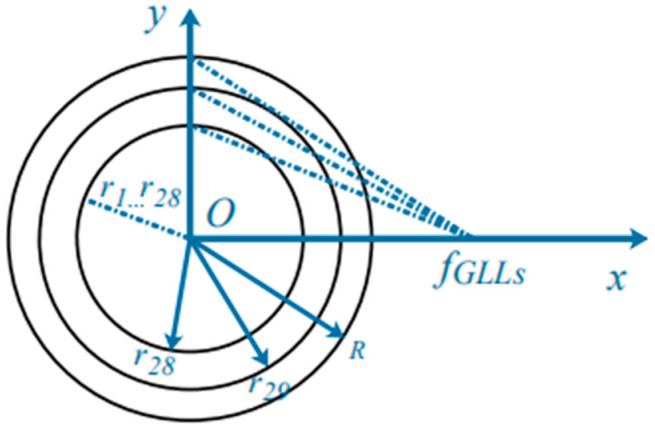
Internal structure diagram of GLLs. We have marked 28, 29, and 30 layers of concentric rings with different refractive indices in the figure. We use *r*_1_…*r*_28_ to represent concentric rings from 1 to 28 layers, *R* represents the maximum radius of GLLs, and *f_GLLs_* represents the focal length of GLLs.

This section will analyze the simulation results. We use DDSCAT to generate a GLLs containing 30 layers with different refractive indices. The internal structure of GLLs is shown in Figure 2. The thickness of each layer is almost the same, and the refractive index of each layer can be calculated by $n_{i}=n_{0}{\left[1+f_{GLLs}^{2}-{\left(r_{0}/R\right)}^{2}\right]}^{1/2}/f_{GLLs}$. In all numerical simulations, the maximum radius R and focal length $f_{GLLs}$ of GLLs are 2 μm and 1.2 a.u., and the wavelength will be introduced separately in each section. The center of mass of GLLs is at (0,0,0), and the plane wave always propagates along +x and is polarized along the y-direction. The reason for choosing these parameters is to ensure correctness (compared with Mie theoretical calculation results) and a better jet effect. The nanoparticles are gold nanorods, and their polarizability is given in Equations (11)–(13). As the calculation framework in this paper is dipole approximation, the volume of nanoparticles must meet specific requirements, and the effective radius of nanorods must meet certain requirements $2\pi a_{eff}≳1$. In this paper, we only discuss the effect of orientation and dielectric constant of nanorod on $F_{x}$, $F_{y}$, and $F_{z}$. Both electric field (a.u.) and optical force (in N) are displayed in the xoy plane and the positions of GLLs are marked with white circles. When the electric field is shown separately, the value is $\left|E\right|/\left|E_{0}\right|$. For convenience, we will use |E| to represent the value. Because the photonic jet is a superposition of the incident and scattering fields on the shadow side of the particle, we must know the ratio of the field in the area of the photonic jet to the incident field. There are two other important parameters for PJ, which are the focal length *f* and the full width at half maxima (*FWHM*) at the focal point. These two parameters describe the distance from the focal point of the PJ to the outer surface of the GLLs and the width where the intensity is half the intensity of the focal point, respectively. They respectively represent the horizontal and vertical scales of the PJ. When the *FWHM* is less than half of the wavelength, the PJ may break the diffraction limit, so these two scales tend to be measured in wavelength λ.

In the following, we first compare the photonic jet of GLLs under the DDA framework with that under the Mie theory framework. Here, we will only show the comparison results of photonic jet with wavelength 0.5 μm and 0.6328 μm. Then we analyzed the influence of the orientation of the nanorods on the optical force, and finally analyzed the influence of the wavelength on the optical force.

This section may be divided by subheadings. It should provide a concise and precise description of the experimental results, their interpretation, as well as the experimental conclusions that can be drawn.

### 3.1. Numerical Validation

**Figure 3:**
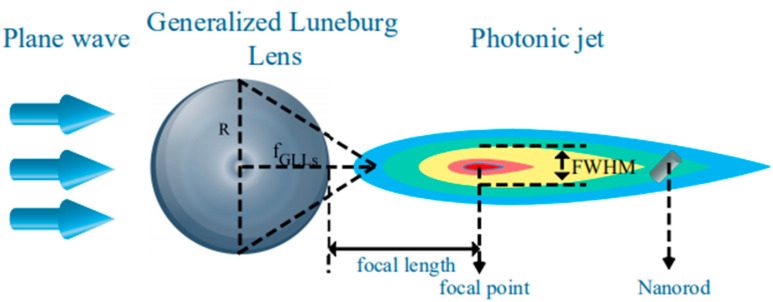
PJ generated by a plane wave illuminating a GLLs. In the figure, plane waves, GLLs, photonic jets, and nanorods and some of their parameters (such as the maximum radius *R* and the focal length *f_GLLs_* of GLLs, the focal point of the photonic jet, the focal length *f* and *FWHM*) are marked respectively. GLLs are composed of 30 layers of concentric rings with different refractive indexes centered on the center of the sphere.

**Figure 4:**
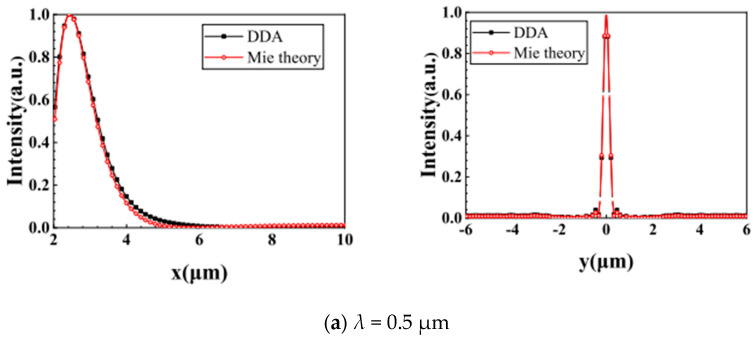

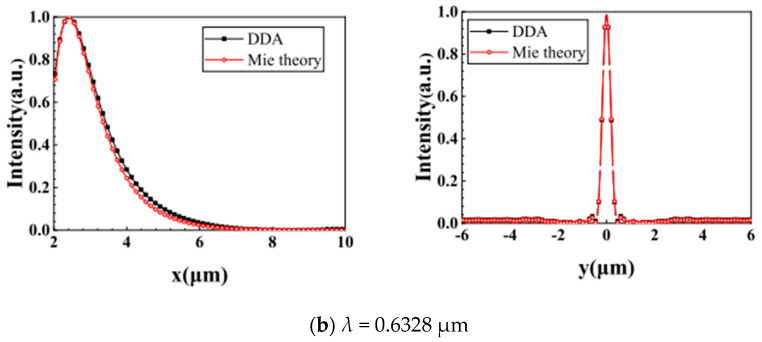
Comparison of theoretical calculation results between DDSCAT and Mie. (**a**,**b**) describe the comparison results of the two methods when the wavelength is 0.5 μm and 0.6328 μm, respectively. The first picture in each row is the intensity distribution along the x-axis through the focal point of the photonic jet. The second picture is also the intensity distribution but along the y-axis through the focal point.

In this section, we calculate the photonic jet generated by a plane wave irradiating a GLLs respectively through DDA and Mie theory and compare the results. The schematic diagram of the PJ irradiating nanorod is shown in Figure 3. DDSCAT and Jan Schäfer [46,47,48,49,50]’s Mie theory software were used, and the two results were normalized by $I_{\mathrm{norm}\;}=\left(I_{i}-I_{\min}\right)/\left(I_{\max}-I_{\min}\right)$ [51], where, $I_{i}$, $I_{\min}$ and $I_{\max}$ are the intensity at any position, the maximum value, and the minimum value, respectively. The error of all comparisons is within one-thousandth. As shown in Figure 4, the wavelengths are 0.5 (Figure 4a) and 0.6328 μm (Figure 4b) respectively, the maximum radius of GLLs is 2 μm (i.e., Mie size parameter q ∼20), and the focal length is 1.2 a.u.. 

### 3.2. Orientation of Nanorods

**Figure 5:**
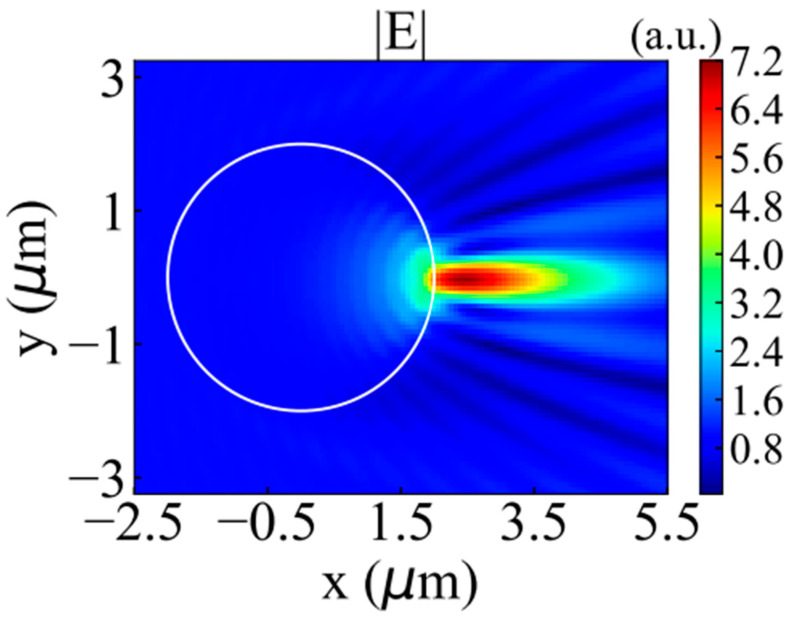
PJ generated by a plane wave with a wavelength of 0.6328 μm irradiating a GLLs.

In this section, we will analyze the influence of nanorod’s orientation on the optical force. As mentioned earlier, the focal length $f_{GLLs}$ and the maximum radius of GLLs are 1.2 and 2 μm. The incident wavelength is 0.6328 μm, the dielectric constant of the gold nanorod is −11.740 + 1.2611*i*, and the volume is 4.2 × 10^−3^ μm^3^. We will first show the electric field of the photonic jet under this set of parameters, as shown in Figure 5. The intensity of the focal point of the photonic jet is 51.7 a.u., the focal length *f* is 0.62 *λ*, and the FWHM is 0.5 *λ*. Next, we will use two subsections to discuss the influence of the orientation $\varphi_{0}$ and $\theta_{0}$ of the nanorods on the optical force.

**Figure 6:**
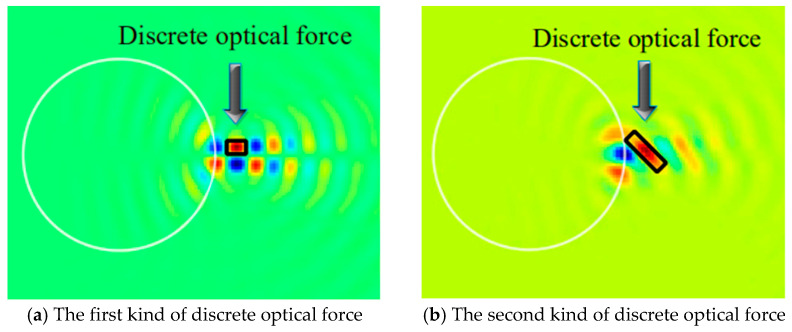
(**a**,**b**) The different kinds of discrete optical forces.

The optical force in this paper has several discrete regions. For convenience, we define the independent region of optical force as discrete optical force, as shown in Figure 6. Figure 6a,b show two kinds of discrete optical forces, respectively.

#### 3.2.1. $\varphi_{0}$ Orientation of Nanorods

**Figure 7:**
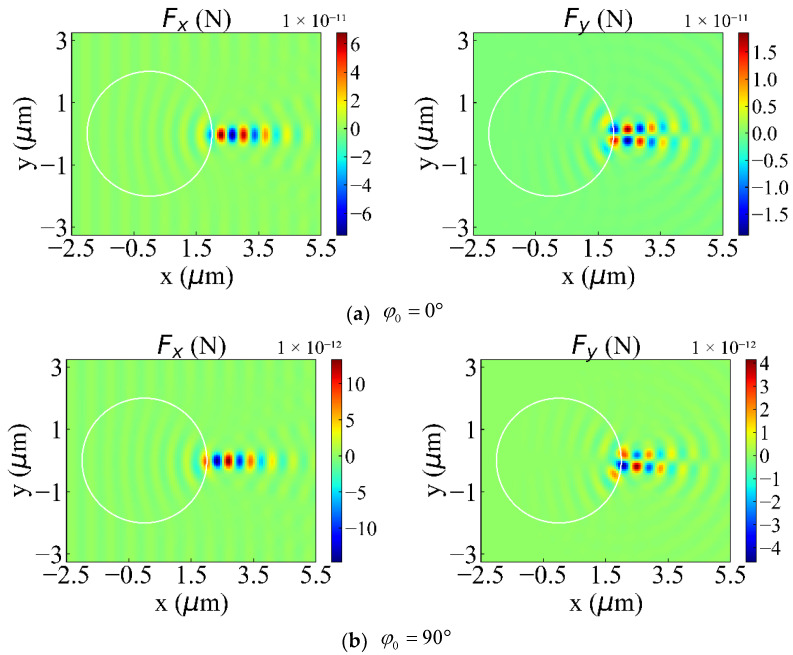

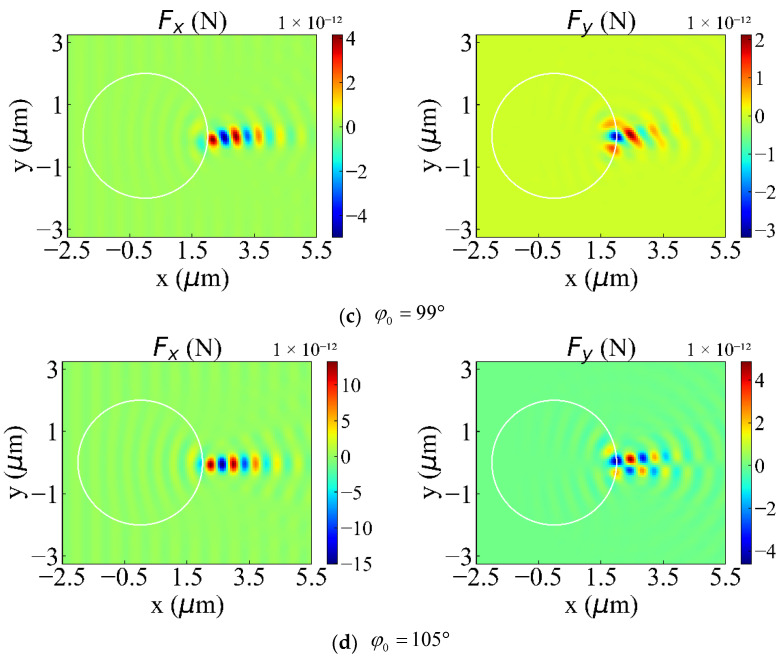
Optical force exerted on a nanorod with different *φ*_0_. Panels (**a**–**d**) show the optical forces *F_x_* and *F_y_* when $\varphi_{0}=0{}^{\circ},\;90{}^{\circ},\;99{}^{\circ},\;\mathrm{and}\;105{}^{\circ}$, respectively.

**Figure 8:**
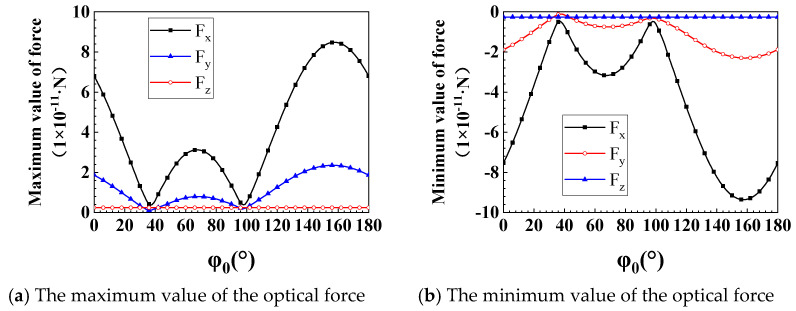
The maximum and minimum values of optical force varies with $\varphi_{0}$. (**a**,**b**) show the change rules of the maximum and minimum values of $F_{x}$, $F_{y}$, and $F_{z}$ in the process of $\varphi_{0}$ increasing from 0° to 180°.

In this section, we will study the influence of the change of $\varphi_{0}$ on the optical force ($\theta_{0}=90{}^{\circ}$). We respectively show $F_{x}$ and $F_{y}$ when $\varphi_{0}=0{}^{\circ},\;90{}^{\circ},\;99{}^{\circ},\;\mathrm{and}\;105{}^{\circ}$. The reason why we choose these angles is because of the unique properties of the discrete optical force at these angles. There are significant differences in the size and shape of these discrete optical forces. Of course, we have calculated the optical forces at all angles. However, the distribution patterns of these optical forces at these four angles is the most representative. We will explain their unique properties one by one. When $\varphi_{0}$ is one of these four cases, the optical force (especially $F_{y}$) has a unique and typical distribution pattern of positive and negative forces. When $\varphi_{0}=0{}^{\circ}$, $F_{y}$ is almost perfectly symmetric with respect to *y* = 0, including the magnitude and scope of positive and negative forces. When $\varphi_{0}=90{}^{\circ}$, the $F_{y}$ in the y>0 area is significantly smaller than the $F_{y}$ in the y<0 area (reversed when $\varphi_{0}=105{}^{\circ}$). When $\varphi_{0}=99{}^{\circ}$, the scope of positive force increases significantly, and the negative force converges on the particle surface. Still, the maximum value of the negative force is higher than that of the positive force. Because of the uniqueness of the optical forces in these four angles, we decided to show them, as shown in Figure 7a–d. When $\varphi_{0}$ is equal to other values, the distribution pattern of positive and negative forces is always similar to the above four cases, so it will not be shown. In increasing $\varphi_{0}$ from 0 to 180 degrees, the change of $F_{x}$ is mainly reflected in the intensity of the force. At the position of the PJ, the optical force is symmetrical about y = 0, and the positive force and the negative force alternately appear. The maximum value of the positive force and the negative force are almost equal, and both have a clear action area. Therefore, when capturing nanorods with different orientations in the light propagation direction (x), the negative force can always be exerted on the nanorods by accurately moving the GLLs to achieve the purpose of capture. *F_y_* has different directions of action on both sides of y = 0, and the positive and negative forces also appear alternately along +x. Therefore, the nanorods oscillate repeatedly on both sides of y = 0. However, when $\varphi_{0}\geq 45{}^{\circ}$, the positive and negative forces on both sides of y = 0 appear asymmetry. The force on one side is more potent than the other side. Such particles may always move to one side in the y-direction. At this time, we can also move GLLs along the x-direction so that the nanorods always receive the same magnitude and opposite optical forces on both sides of *y*. This allows the nanorods to be in a state of dynamic equilibrium and to be stably captured in the y-direction. Figure 8 shows the variation of the maximum and minimum values of $F_{x}$, $F_{y}$, and $F_{z}$ with $\varphi_{0}$. As shown in Figure 8a,b we calculate the change rules of the maximum and minimum values of $F_{x}$, $F_{y}$, and $F_{z}$ in the process of $\varphi_{0}$ increasing from 0° to 180°. Since the light propagates in the x direction and $\theta_{0}=90{}^{\circ}$, the radius of the bottom surface of the nanorod is small, so the intensity of $F_{z}$ is relatively tiny compared to $F_{x}$ and $F_{y}$. Through research, it can be found that the maximum and minimum values of $F_{x}$ and $F_{y}$ have precisely the same trend with the increase of $\varphi_{0}$. The optical force decreases first, reaching a minimum at 39°. Then increase, get a local maximum at 69°, and drop immediately. When $\varphi_{0}=99{}^{\circ}$, it comes the local minimum value and increases. Reach the maximum value when $\varphi_{0}=159{}^{\circ}$. In summary, different $\varphi_{0}$ will cause the force on nanorods to be very different, so we can propose targeted solutions for particles with different $\varphi_{0}$ to achieve stable capture.

#### 3.2.2. $\theta_{0}$ Orientation of Nanorods

**Figure 9:**
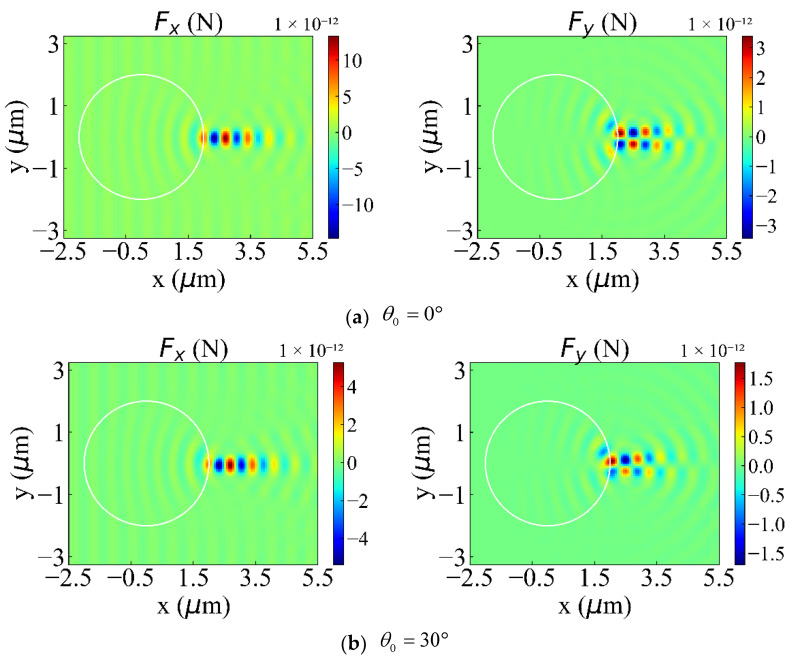

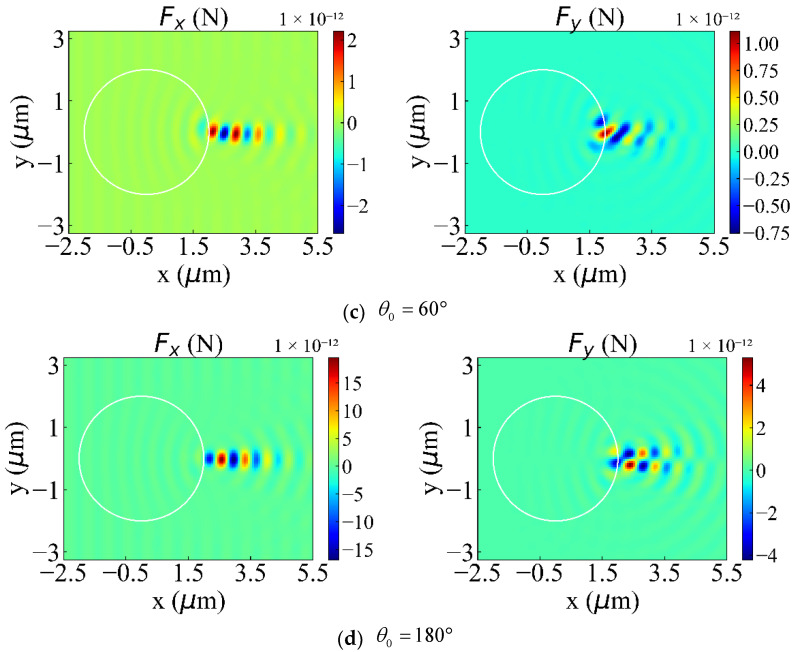
Optical force exerted on a nanorod with different *θ*_0_. Panels (**a**–**d**) show the optical forces *F_x_* and *F_y_* when $\theta_{0}=0{}^{\circ},\;30{}^{\circ},\;60{}^{\circ},\;\mathrm{and}\;180{}^{\circ}$, respectively.

**Figure 10:**
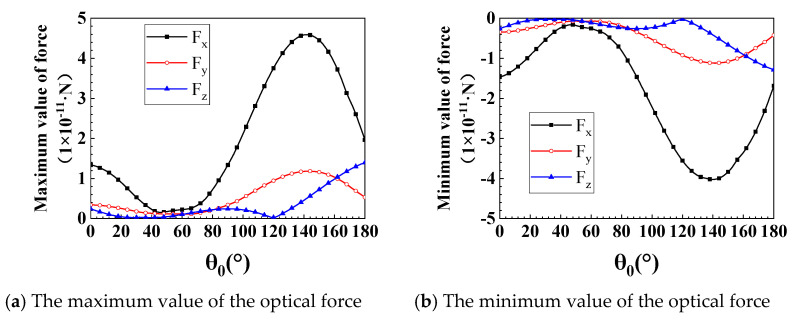
The maximum and minimum values of optical force varies with $\theta_{0}$. (**a**,**b**) show the change rules of the maximum and minimum values of $F_{x}$, $F_{y}$, and $F_{z}$ in the process of *θ*_0_ increasing from 0° to 180°.

In this section, we will discuss the influence of $\theta_{0}$ on optical forces ($\varphi_{0}=45{}^{\circ}$). As shown in Figure 9a–d, we offer $F_{x}$ and $F_{y}$ at $\theta_{0}=0{}^{\circ},\;30{}^{\circ},\;60{}^{\circ},\;\mathrm{and}\;180{}^{\circ}$, respectively. Similarly, these angles were chosen to demonstrate the unique properties of discrete optical forces and the distribution patterns of positive and negative forces. The distribution pattern of positive and negative forces determines the direction of force and the way to capture the particles. When $\theta_{0}=0{}^{\circ}$, the discrete optical force of $F_{y}$ is almost perfectly symmetric with respect to y = 0. When $\theta_{0}=30{}^{\circ}$, the discrete optical force of $F_{y}$ in the y<0 area is smaller than the $F_{y}$ in the y>0 area. When $\theta_{0}=60{}^{\circ}$, the scope of negative force increases significantly, and the positive force converges on the particle surface. Still, the maximum value of the positive force is higher than that of the negative force. When $\theta_{0}=180{}^{\circ}$, the range of negative force is greater than that of positive force, but the maximum value of negative force is smaller. Because of the uniqueness of the optical forces in these four angles, we decided to show them, as shown in Figure 9a–d. When $\theta_{0}$ is other values, the distribution pattern of optical force is similar to the above four situations, and the repeated content will not be shown in this article. $\theta_{0}$ in 0° − 42°, $F_{x}$ and $F_{y}$ are almost all symmetric with respect to y = 0, with only variations in the intensity of the force. Still, the range of $F_{y}$ changes significantly around 30°, 60° and 180°, as shown in Figure 9b–d. $F_{x}$ is not symmetric about y = 0. The range of the $F_{x}$’s negative force is significantly increased. The wide distribution of negative forces allows particles to be captured more easily. Figure 10 shows the variation of the maximum and minimum values of $F_{x}$, $F_{y}$, and $F_{z}$ with $\theta_{0}$. As shown in Figure 10a,b, we studied the maximum values of the positive and negative forces of $F_{x}$, $F_{y}$, and $F_{z}$ in the range of $0{}^{\circ}\leq \theta_{0}\leq 180{}^{\circ}$. The numerical results show that the maximum values of $F_{x}$ and $F_{y}$ decrease first, then increase and finally decrease again with the increase of $\theta_{0}$. When $\theta_{0}=120{}^{\circ}$, $F_{z}$ is a local minimum value. Otherwise, the trend of $F_{z}$ increasing with $\theta_{0}$ is basically the same as that of $F_{x}$. The maximum value of positive force and negative force are almost equal. Under the same parameters, the area where the maximum positive force and the negative force are located in the photon ejection is fixed. By moving the GLLs, the nanorods can be accurately captured.

### 3.3. Dielectric Constant

**Figure 11:**
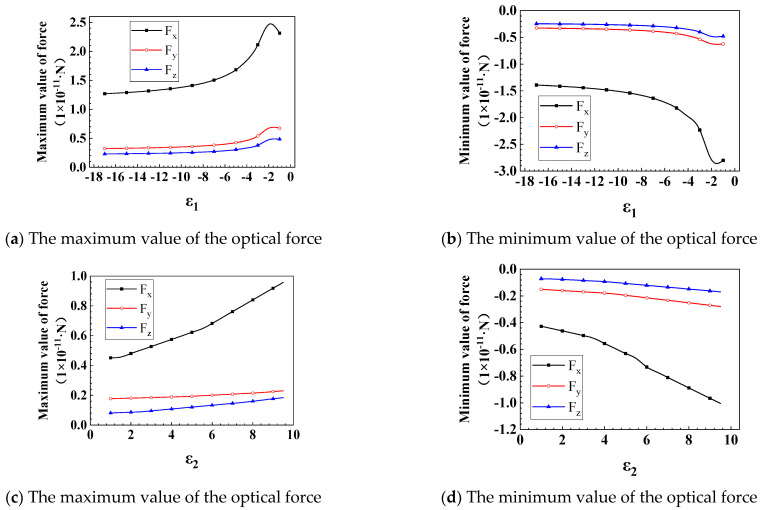
The maximum and minimum values of optical force varies with *ε_m_*. Panels (**a**–**d**) describe the effects of the real and imaginary parts of the nanorod’s dielectric constant on the maximum and minimum values of optical force, respectively.

In this section, we discuss the effects of the real ($\varepsilon_{1}$) and imaginary ($\varepsilon_{2}$) parts of the nanorod’s dielectric constant on the optical force, as shown in Figure 11. The $f_{GLLs}$ and *R* of GLLs are 1.2 and 2 μm. The wavelength is 0.6328 μm, and the volume of the nanorod is 4.2 × 10^−3^ μm^3^. In Figure 11a,b we show the maximum and minimum optical force changes with the real part ($\varepsilon_{1}$) of the dielectric constant when the imaginary part $\varepsilon_{2}$ of the nanorod’s dielectric constant is 1, respectively. From the figures, we can find that the optical force first increases and then decreases with the increase of $\varepsilon_{1}$. When $\varepsilon_{1}=-2$, the optical force has the local maximum value. From Figure 11c,d, we can find that as $\varepsilon_{2}$ increases, the optical force gradually increases.

## 4. Conclusions

In this paper, we studied the optical forces $F_{x}$ and $F_{y}$ exerted by a photonic jet on nanorods under the framework of the dipole approximation. The PJ is generated by a plane wave irradiating a GLLs, and the numerical result is calculated by DDSCAT. We divided GLLs into 30 layers of concentric rings with different refractive indices. We compare the calculation results of DDSCAT with the results of the Mie theory to validate the PJ’s electric field. Then we use the PJ to illuminate the nanorods and study the optical forces *F_x_* and *F_y_* on the nanorods. Of course, the size of nanorods must satisfy the Rayleigh approximation. The effects of the orientation and dielectric constant of nanorods on the optical force are investigated. The angle between the axis of the nanorod and the z-axis is represented by $\theta_{0}$, and the angle between the projection of the axis on the xoy plane and the x-axis is defined by $\varphi_{0}$. We first discuss $\varphi_{0}$’s effect on $F_{x}$ and $F_{y}$ by making $\theta_{0}=90{}^{\circ}$ (the axis of the nanorods is in the xoy plane). We find that $F_{x}$ is always symmetric about the y-axis, and $F_{y}$ is not symmetric about the y-axis, only around $\varphi_{0}=90{}^{\circ}$ and $\varphi_{0}=105{}^{\circ}$. In contrast, the intensity of $F_{z}$ can be ignored since the maximum value of the positive and negative optical forces are almost equal. It is necessary to constantly adjust the position of the GLLs when capturing these oriented nanorods so that the nanorods are always in the area of negative or positive forces. Then we investigate the effect of $\theta_{0}$ on optical force when $\varphi_{0}=45{}^{\circ}$. The numerical results show that $F_{x}$ is asymmetric about the y-axis near $\theta_{0}=45{}^{\circ}$ and 60°, and the area of the negative force of $F_{y}$ increases significantly (including $\theta_{0}=180{}^{\circ}$), and the positive force converges on the surface of GLLs. Similarly, the maximum values of the positive and negative optical forces are equal. Due to the wider distribution of negative forces at certain angles, it is easier to adjust GLLs to capture nanorods. Finally, we discussed the effect of the dielectric constant of the nanorods on the optical force. Under the same conditions, a larger dielectric constant generally leads to a powerful optical force. In this paper, the optical force exerted by the photonic jet on elongated particles in the form of nanorods instead of spherical nanoparticles with different orientations and materials (dielectric constant) is studied. The numerical results describe the direction and magnitude of the optical force, which makes it possible to directionally manipulate the nanorods. Note that the presented results will be valid not only for GLLs, but also for other particles [52] that form a photonic jet with similar characteristics. These results are expected to provide theoretically support for the manipulation of nanorods and the arrangement of nanoarrays.

## Data Availability

Some or all data, models, or code generated or used during the study are available in a repository or online in accordance with funder data retention policies (Provide full citations that include URLs or DOIs.)

## Abbreviations

The following abbreviations are used in this manuscript:

PJ	Photonic jet
GLLs	Generalized Luneburg Lens
DDA	Discrete Dipole Approximation
FWHM	Full Width at Half Maxima
